# Oral Nutritional Supplements Reduce Body Weight Loss after Gastrectomy in Patients with Gastric Cancer: A Systematic Review and Meta-Analysis of Randomized Controlled Trials

**DOI:** 10.3390/nu15183924

**Published:** 2023-09-10

**Authors:** Mijoo Choi, Jong-Yeup Kim, Hyun-Hi Kang, Eunju Park, Sung Ryul Shim

**Affiliations:** 1 Department of Food and Nutrition, Kyungnam University, Changwon 51767, Republic of Korea; mijoo@kyungnam.ac.kr; 2 Department of Otorhinolaryngology-Head and Neck Surgery, College of Medicine, Konyang University, Daejeon 35365, Republic of Korea; jykim@kyuh.ac.kr; 3 Department of Biomedical Informatics, College of Medicine, Konyang University, Daejeon 35365, Republic of Korea; 4 Department of Food and Nutrition Care Service, Gyeongsang National University Changwon Hospital, Changwon 51472, Republic of Korea; ghqlsdl@gnuh.co.kr

**Keywords:** dietary supplements, stomach neoplasms, gastrectomy, body weight

## Abstract

This systematic review and meta-analysis aimed to summarize the effects of oral nutritional supplements (ONSs) on body weight loss (BWL) after gastrectomy. A systematic search was conducted across the PubMed, Cochrane, and Embase databases through May 2023. The study inclusion criteria were as follows: (1) studies on interventions including ONSs after gastrectomy in patients with gastric cancer; (2) studies in which comparisons were specified according to standard, regular, or usual postoperative diets; and (3) randomized controlled trial studies including outcomes measured as mean differences in BWL. The data were pooled using the random-effects model and expressed as mean differences with 95% confidence intervals (CI). Based on data from seven studies including 1743 patients (891 for ONSs and 852 for the control), the overall pooled mean difference was 0.848 (95% CI: 0.466 to 1.230) and the Higgins *I*^2^ value was 86.0%. This systematic review and meta-analysis is the first study to show that ONSs are significantly associated with reducing BWL, compared with standard diets, after gastrectomy in patients with gastric cancer. Furthermore, we found that ONSs were more effective in patients with lower nutritional kilocalorie intake after gastrectomy.

## 1. Introduction

Every year, approximately one million people are diagnosed with gastric cancer worldwide, and approximately 1.1 million new cases and 770,000 deaths were reported in 2020. Gastric cancer is the fifth most common cancer and the fourth most common cause of cancer-related death [1]. Despite major advances in medical treatment, including chemotherapy and immunotherapy, surgical resection is considered the only treatment for early and advanced gastric cancer [2,3]. However, owing to decreased stomach volume, reduced food intake, and gastrointestinal symptoms, most patients with gastric cancer suffer from severe malnutrition [4]. Body weight loss (BWL) is common in patients who have undergone gastrectomy for gastric cancer, and it manifest in patients typically losing 10–20% of their preoperative weight [5]. BWL increases morbidity and mortality, worsens chemotherapy tolerance, and ultimately reduces survival rates [6].

Oral nutritional supplements (ONSs) come in liquid, cream, or powdered form that can be added to drinks or foods, and they are developed to provide energy and nutrient-dense solutions. ONSs are widely recognized as one of the most important forms of nutritional support for cancer and postoperative patients [7]. However, a consensus has not yet been reached on whether ONSs can effectively reduce BWL in patients with cancer undergoing surgery [8,9]. In addition, rigorous and analytical studies of individual covariates, particularly the intake of calories, gender, age, and TNM stages, have not been reported to date in systematic reviews and meta-analyses. Nevertheless, recently published well-designed randomized controlled trials (RCTs) have reported this effect [10,11].

Therefore, this systematic review and meta-analysis aimed to evaluate the effect of ONSs on nutritional status after gastrectomy in patients with gastric cancer and to identify associated risk factors as moderating variables.

## 2. Materials and Methods

This study adhered to the reporting guidelines outlined in the Preferred Reporting Items for Systematic Reviews and Meta-Analyses (PRISMA) reporting guidelines [12]. The study protocol related to this research was registered on PROSPERO with the registration number CRD42023431228. This study used publicly available data from the PubMed, Embase, and Cochrane Library databases and others, and it did not include human participant research. As Per 45 CFR §46.102(f), this study was not submitted for institutional review board approval and did not require informed consent.

### 2.1. Data Sources and Literature Searches

A thorough search of the PubMed, Embase, and Cochrane databases was performed using medical subject headings (MeSH) terms and text keywords from the start of the databases to May 2023: PubMed and Cochrane [(“Stomach Neoplasms”[Mesh] OR “Stomach Neoplasms”[tiab] OR “Gastric Cancer”[tiab] OR “Gastric Neoplasm*”[tiab] OR “Gastrectomy”[Mesh] OR “Gastrectomy”[tiab]) AND (“Dietary Supplements”[Mesh] OR “Dietary Supplements”[tiab] OR “Oral nutritional supplements”[tiab]) AND (“Body Weight”[Mesh] OR “Body Weight”[tiab])], Embase [(‘stomach tumor’/exp OR ‘stomach tumor’:ti,ab OR ‘stomach neoplasm’:ti,ab OR ‘gastric cancer’/exp OR ‘gastric neoplasm’:ti,ab OR ‘gastrectomy’/exp OR ‘gastrectomy’:ti,ab) AND (‘oral nutritional supplement’/exp OR ‘oral nutritional supplement’:ti,ab OR ‘dietary supplement’:ti,ab) AND (‘body weight’/exp OR ‘body weight’:ti,ab)] (Table S1). PubMed and Cochrane use the same MeSH, and in the related terms search, if the disease and outcome measure did not match exactly in the MeSH, we expanded the search to higher-level concepts. In Embase, we searched based on Emtree and also expanded to the most similar term if the terms did not match. The subject headings and text keywords were related to BWL in patients receiving ONSs after gastrectomy. The search terms were categorized using Boolean operators (e.g., AND, OR, and NOT). Only RCTs were included in this meta-analysis. This search was conducted regardless of language or study type. Two independent researchers (SR Shim and MJ Choi) supplemented the search by manually examining trial databases and reference lists to identify additional relevant studies. To ensure consistency among the researchers conducting the search and the accuracy of the search, we held at least two pre-study meetings before establishing the literature search strategy and completed 10 h of accredited training in targeted literature searching from a professional organization. The search researchers were all Ph.D.s and had at least five years of experience working in a professional research organization.

### 2.2. Study Selection

The study inclusion criteria were as follows: (1) studies including patients diagnosed with gastric cancer; (2) studies on interventions including ONSs after gastrectomy; (3) studies in which comparisons were performed according to standard, regular, or usual postoperative diet categories; and (4) RCT studies including outcomes measured as mean differences in BWL. To ensure data accuracy and relevance, duplicate publications and articles without original data (such as case reports, abstracts only, review articles, editorials, and letters) were excluded. Furthermore, studies lacking comparison groups were excluded from the analysis. The titles, abstracts, and full-text articles were evaluated independently by two investigators (SR Shim and MJ Kim), following the predetermined inclusion and exclusion criteria. Data extraction was performed by the authors using dedicated data extraction forms, and article inclusion was confirmed through a collaborative evaluation discussion involving all investigators. To ensure the accuracy and integrity of the meta-analysis, references and data from each included study were thoroughly examined to eliminate any potential overlap.

### 2.3. Data Extraction

The basic details of the studies (first author, publication year, country, study design, ONS nutrient type, controls, and treatment duration), patient characteristics (number of patients, age, female ratio, kilocalorie per day consumption, and TNM stage), and technical aspects such as inclusion and exclusion criteria, as well as treatment details, were extracted from the included articles using a predetermined data extraction form. If a study included multiple treatment periods, the effect size was calculated for each period. In cases where the studies did not report standard deviations, a combined standard deviation for the two groups was estimated. The final meta-analysis included only studies that provided comprehensive information.

### 2.4. Statistical Analysis

To measure BWL, the mean differences along with their 95% confidence intervals (CIs) were calculated for continuous variables. A random-effects model analyzed using a restricted maximum-likelihood (REML) estimation was used to obtain the pooled overall mean differences and 95% CIs for outcomes [13]. Statistical heterogeneity was assessed by Cochran’s Q test and Higgins’ *I*^2^ values. For Cochran’s Q, a value of *p* < 0.1 was considered to indicate statistically significant heterogeneity. If either Cochran’s Q statistics (*p* < 0.1) or *I*^2^ value (>50%), this indicated the existence of significant heterogeneity between the studies. For example, *I*^2^ values of 0% to 40% might not be important; 30% to 60% may represent moderate heterogeneity; 50% to 90% may represent substantial heterogeneity; and 75% to 100% may represent considerable heterogeneity [14].

A meta-regression analysis was performed for the moderators comprising continuous variables such as the number of patients, age, proportion of females, and treatment duration. Additionally, a meta-ANOVA was conducted for categorical variables, including TNM stage, daily kilocalorie consumption, and country. TNM stage in individual studies was categorized as a dichotomous variable (≥50% versus <50% for stage III and above). The REML estimator was used to evaluate the variance of true effects to analyze potential moderators. A two-sided *p*-value ≤ 0.05 or the absence of a null value (mean difference = 0) within the 95% CIs was considered significant. This analysis was conducted using R software (version 4.2.1; R Foundation for Statistical Computing) [14].

### 2.5. Assessment of Potential Publication Bias

To assess the potential presence of publication bias, a funnel plot was created. The funnel plot utilized the standard error as a measure of study size and plotted the mean differences between the ONS and control groups. In the absence of publication bias, the studies typically demonstrated a symmetrical distribution based on the combined effect size. To further evaluate publication bias, we performed Egger’s linear regression test, as well as the Begg and Mazumdar rank correlation tests [14]. The two commonly employed tests aim to quantify the extent of bias depicted in the funnel plot. Begg and Mazumdar’s rank correlation test assesses the rank correlation between standardized effect sizes and their associated standard errors. If this test shows no significance, it indicates a lack of publication bias. On the other hand, Egger’s linear regression method evaluates the linear regression of the intervention effect estimate against its standard error, incorporating inverse variance weighting. In Egger’s test, the null hypothesis is that the linear regression model’s slope is zero. A failure to reject this null hypothesis suggests an absence of publication bias. Unlike Begg and Mazumdar’s test, Egger employs actual effect size values and their precision instead of ranks.

### 2.6. Quality Assessment

The Cochrane Collaboration risk-of-bias (RoB) 2.0 tool was used to assess the risk of bias and the methodological quality of the RCTs. Five domains were assessed, and each domain was assigned a risk of bias rating of high, low, or unclear. If all domains received a “low” rating, the overall risk of bias was considered low. If at least one domain received a “some concerns” rating, it indicated some concerns. However, if at least one domain received a “high” rating or more than two domains received a “some concerns” rating, the overall risk of bias was considered high [15].

## 3. Results

### 3.1. Study Selection

**Figure 1:**
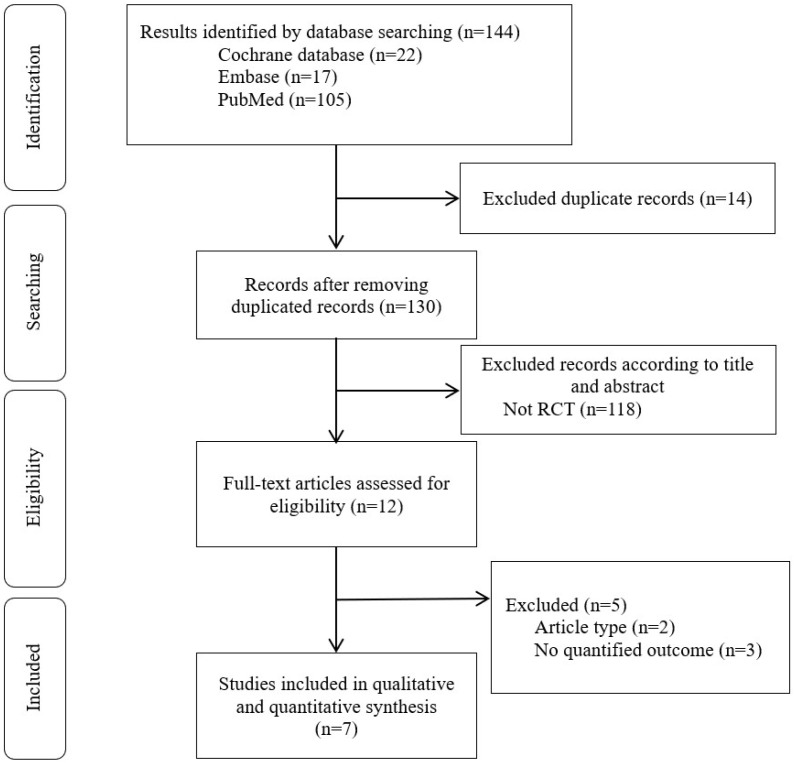
PRISMA flowchart.

The initial search yielded 144 articles from different electronic databases: PubMed (n = 105), Cochrane (n = 22), and Embase (n = 17). Of these, 14 studies were excluded due to either containing overlapping data or appearing in multiple databases. Following title and abstract screening, 118 studies were either eliminated because they were trial registrations or consisted solely of abstracts. Among the 12 full-text articles assessed, 5 were further excluded due to them not being original articles (n = 2) or lacking quantified outcomes (n = 3). Ultimately, seven studies [10,11,16,17,18,19,20] met the selection criteria for qualitative and quantitative analyses (Figure 1 and Table 1). In the study without a standard deviation, the estimate of the pooled standard deviation of the two groups (before/after) was applied. However, we excluded cases that could not be calculated due to a lack of precise information or where results were only presented in graphs. In addition, if the final outcome indicator and study design did not match, we judged the information to be inaccurate and did not include it in the final list of eligible studies.

### 3.2. Outcome Findings 

**Figure 2:**
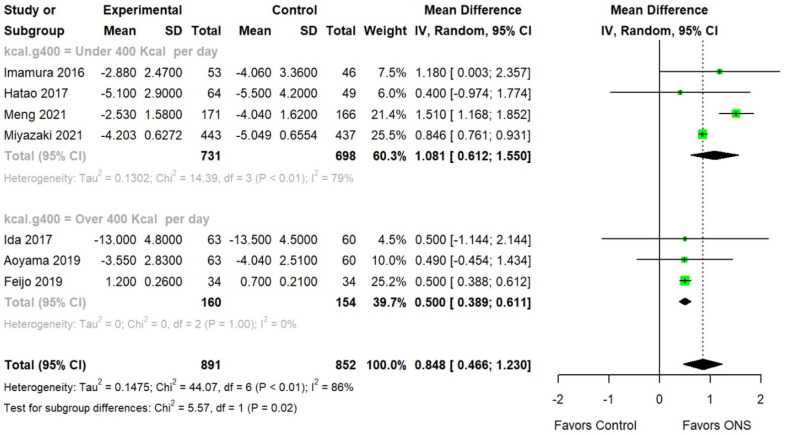
Forest plots of body weight between the oral nutritional supplements and control groups. Imamura (2016) [20], Hatao (2017) [18], Ida (2017) [19], Aoyama (2019) [16], Feijo (2019) [17], Meng (2021) [10], Miyazaki (2021) [11].

The pooled mean difference in BWL in the ONS group compared with the control groups at 3 months was 0.848 (95% CI: 0.466, 1.230), which was statistically significant. The heterogeneity test showed significance at *p* < 0.001, and the Higgins’ *I*^2^ value was 86.0%. For the evaluation of calorie intake which has an impact on the improvement of BWL, we also conducted a subgroup analysis. The pooled mean difference in BWL in the ONS group compared with the control groups of under 400 kilocalories per day was 1.081 (95% CI: 0.612, 1.550), which was statistically significant. The heterogeneity test showed significance at *p* < 0.001, and the Higgins’ *I*^2^ value was 79.0%. The pooled mean difference in BWL in the ONS group compared with the control groups of over 400 kilocalories per day was 0.500 (95% CI: 0.389, 0.611), which was also statistically significant. The heterogeneity test showed significance at *p* = 1.000, and the Higgins’ *I*^2^ value was 0.0%. (Figure 2).

We conducted an additional analysis by treatment duration, with a mean difference of 0.803 (95% CI: 0.412, 1.195) at 6 months and 0.718 (95% CI: 0.278, 1.157) at 12 months, a slight reduction in the ONS effect but still statistically significant.

### 3.3. Moderator Analysis

This study explored the potential moderating roles of specific variables through meta-regression and meta-analysis of variance models (Table 2). We found statistically significant differences in daily kilocalorie consumption (*p* < 0.018), with the <400 kcal group having a significantly higher body weight than the >400 kcal group. The group with a TNM stage above III had a significantly lower value at 0.500 (95% CI, 0.388, 0.611) than the group with a TNM stage below III did at 1.046 (95% CI, 0.603, 1.488) (*p* = 0.019). In addition, significant differences were observed according to country (*p* < 0.001), with Asian countries (Japan and China) having a higher body weight at 0.842 (95% CI; 0.758, 0.926) and 1.510 (95% CI; 1.168, 1.852) than non-Asians countries (Brazil) at 0.500 (95% CI; 0.388, 0.612). A higher female rate was associated with greater BWL (*β* = −2.505), but due to the small number of seven studies and the insufficient sample size, the results were not statistically significant (*p* = 0.689). No significant differences were observed among the remaining covariates.

### 3.4. Publication Bias

The statistical methods employed to detect publication bias or small-study effects are shown in Figure S1. The mean differences showed visually symmetric graphics in the funnel plots, and the Egger’s linear regression test (*p* = 0.788) and Begg and Mazumdar rank correlation tests (*p* = 0.881) suggested no evidence of publication bias or small-study effects in this meta-analysis. With one study on the left and one on the right outside the funnel and three on the bottom left and two on the right inside the funnel, there was no significant asymmetry based on visual judgment. However, half of the studies are located at the bottom of the funnel, which suggests that the accuracy of the studies due to high standard error may be lacking and should be examined further in the quality assessment.

### 3.5. Quality Assessment

**Figure 3:**
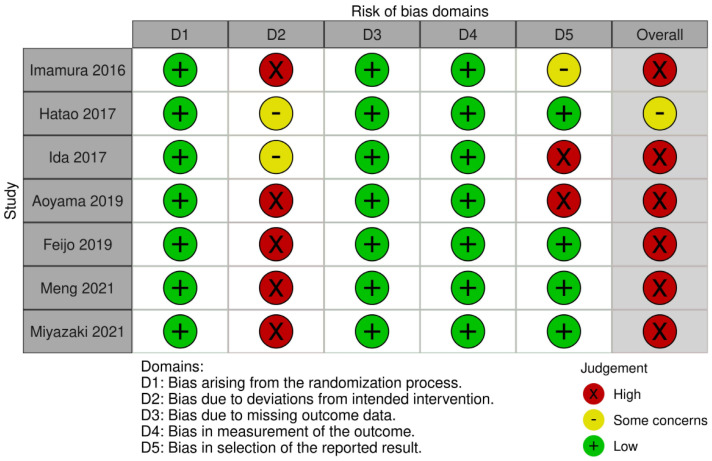
Risk of bias 2.0 assessment. Imamura (2016) [20], Hatao (2017) [18], Ida (2017) [19], Aoyama (2019) [16], Feijo (2019) [17], Meng (2021) [10], Miyazaki (2021) [11].

Seven studies were evaluated using the five RoB 2.0 domains to determine the risk of bias. In D1 (randomization process), all studies were rated as “low”. In D2 (deviations from intended intervention), five studies were rated as “high” and two studies were rated as “some concerns”. In D3 and D4, seven studies were rated as “low”. In D5, two studies were rated as “high”. Aoyama 2019 [16] was rated as high risk at D2 and D5. In fact, ROB 2.0 is basically a system that judges the entire individual study as high risk even if there is only one high risk in the domain-specific quality assessment. In particular, Hatao 2017 [18] and Ida 2017 [19] were not completely high risk at D2, but given the context of the studies, it was deemed unreasonable to assign a low risk of bias. Thus, we ranked these as some concerns. Most studies on postoperative ONS administration were not blinded [10,11,16,17,20]; therefore, the overall risk of bias was rated as “high”, except for one study [18] (Figure 3).

## 4. Discussion

This systematic review and meta-analysis is the first study to show a significant association between ONS and BWL reduction, compared with standard diets, after gastrectomy in patients with gastric cancer. Furthermore, we found that ONSs were more effective in patients with lower nutritional calorie intake after gastrectomy.

According to the European Society for Parenteral and Enteral Nutrition (ESPEN) guidelines, enteral nutrition is indicated in surgical patients, especially those who have undergone upper gastrointestinal surgery [21]. Because BWL can affect the dose intensity of chemotherapy and the survival of patients with gastric cancer after gastrectomy, healthcare professionals are paying attention to short- and medium-term BWL after gastrectomy [22]. Although some studies on ONSs have been controversial regarding BWL [8,9], the results of the present study add to the latest findings and are highly reliable owing to the relatively large patient population, indicating that ONS administration for BWL after gastrectomy is critical to patient survival and improved nutritional status. Malnutrition in cancer patients, which can lead to poor prognosis, requires vigilant attention, particularly in cases where patients are capable of eating but are at risk of or are experiencing malnutrition [23,24]. Nutritional interventions include strategies such as diet modification, addressing symptoms and barriers to food intake, and providing ONSs, and this study shows that appropriate nutritional interventions with ONSs are effective in preventing such malnutrition. In fact, a postoperative BWL study of patients with gastric cancer found that they lost about 4.6% of their body weight from preoperative gastrectomy to discharge, with the greatest weight loss at about 8.1% at 6 months post discharge and they had not regained their preoperative weight by 1 year post discharge [25]. Although subjective postoperative intake gradually improved during the post-discharge period, patients still reported eating 70% or less of their preoperative intake one year after discharge, confirming weight loss due to insufficient intake [25]. Thus, insufficient postoperative intake is one of the main causes of malnutrition [26], suggesting the need for adequate nutritional supplementation. 

The ESPEN guidelines also recommend the administration of ONSs as part of routine clinical care for patients with gastric cancer before surgery or during chemotherapy treatment because they are at nutritional risk and their nutritional intake is limited after surgery. The minimum recommendation for ONSs is 400 kcal per day to complement daily meals [27,28]. However, a study [19] failed to demonstrate the effectiveness of a 600 kcal/d ONS diet rich in eicosapentaenoic acid (EPA) compared with a regular diet after total gastrectomy for gastric cancer. The researchers concluded that the negative result was observed because ONS administration caused a decrease in the oral intake of the regular diet. This is due to functional changes in the stomach, which makes it less able to digest and absorb water; therefore, a large concentration or large calorie intake is not necessarily effective for BWL, as it can lead to hyperosmolar diarrhea, a postoperative complication of hyperosmolarity in the intestines [29,30]. The subgroup analysis of our study also supported these findings that the group with a caloric intake of <400 kcal demonstrated significantly lower BWL than the group with a caloric intake of >400 kcal. 

When comparing differences in nutritional status according to the TNM stages, several studies have demonstrated that patients with lower TNM stages have significantly higher nutritional intake and body weight, compared with patients with higher TNM stages [31,32,33]. Yoon et al. reported the ratio of daily intake to daily requirement in Korea by TNM stage as 1.37 for stage 2, 0.97 for stage 3, and 0.88 for stage 4, indicating a statistically significant decrease in nutritional status with increasing severity [33]. Additionally, the relative weights according to the TNM stage were within the normal range in 67.2% of patients in stages 1 and 2 and 32.8% of patients in stages 3 and 4 [33]. Notably, patients with higher TNM stages had significantly lower intake of calories, carbohydrates, and vitamin B1, supporting the notion that poorly nourished individuals may have higher intraoperative complication and mortality rates than the general population [34,35]. These findings are consistent with the results of our study that, in the lower TNM stage group, where the disease status was relatively mild, patients were more likely to maintain a normal weight range due to adequate nutritional intake. As a result, it is anticipated that reducing TNM staging and increasing caloric intake could influence nutritional recuperation and the reversal of weight loss, consequently leading to substantial declines in complication frequencies and mortality rates.

The quality of the studies in this study was low (high risk of bias) because ROB 2.0 can only make a final judgment of high risk even if there was one high risk by default. Regarding postoperative nutrition, the blinded domain was judged to be high risk because patients cannot know their regular diet and ONSs. However, the studies were all RCT studies with high grades D1 (randomization process), D3 (missing outcome data), and D4 (measurement of the outcome), so it can be said that the quality level was compliant for behavioral treatments such as surgery and dietary therapy.

This systematic review has several limitations. First, this study included only RCTs, which may have limited the number of observational studies. In addition, due to the nature of the study, the patients and health professionals were not blinded, which is likely to increase the risk of deviation from the intended intervention bias. Nevertheless, we believe that the use of RCTs to calculate the pooled overall effect sizes ensures high study quality. Second, the control groups in the included studies varied (regular diet, standard diet, or usual postoperative diet group); however, we did not expect a significant difference in the nutritional composition of the diets. Third, although we calculated the effect size by treatment duration, it was limited by the fact that only Miyazaki et al. (2021) reported values at 3, 6, and 12 months [11]. However, a study reported that ONSs reduced BWL not only 6-8 weeks after gastrectomy but also up to 1 year afterward [36]; therefore, we believe that the therapeutic effects of ONSs were generally observed in other studies. Additional studies with different treatment durations are required to determine the actual impact of ONSs. Fourth, most of the included studies were conducted in Asia, particularly Japan, which has the disadvantage of not being able to characterize ethnicity across the board. If more studies are conducted in the West in the future, it will be necessary to include all of them in a comprehensive effect size determination. Despite these limitations, the results of this meta-analysis may provide quantitative information for understanding the association between body weight and gastrectomy for gastric cancer.

In conclusion, the findings of this systematic review and meta-analysis suggest that administration of ONSs after gastrectomy in patients with gastric cancer significantly reduces BWL, compared with regular diet intake, and this finding has not been shown in previous meta-analyses. Healthcare professionals should consider these findings, especially for patients with low nutritional intake.

## Figures and Tables

**Table 1:** Characteristics of the included studies.

Study	Country	Study Design	Disease	Average Age (Years)	No. of Patients (% Female)	Kcal Consumption Per Day	ONSs Nutrient	Controls	Duration (Months)	TNM Stage (%)
Imamura 2016 [20]	Japan	RCT	Gastric Cancer	66.15	99 (29.75)	300	ONSs	Regular diet	2	I (60.3), II (21.6), III (17.2), IV (0.9)
Hatao 2017 [18]	Japan, Taiwan	RCT	Gastric Cancer	64.7	113 (38.9)	400	ONSs	Usual postoperative diet	3	I (53.1), II (22.1), III (24.8), IV (0)
Ida 2017 [19]	Japan	RCT	Gastric Cancer	65.35	123 (27.6)	600	ONS plus EPA (2.2 g/d)	Standard diet	3	I (40), II (32), III (29), IV (0)
Aoyama 2019 [16]	Japan	RCT	Gastric Cancer	65.35	123 (27.6)	600	ONSs plus EPA (2.2 g/d)	Standard diet	3	I (22.8), II (18.7), III (18.7), IV (39.8)
Feijo 2019 [17]	Brazil	RCT	Gastric Cancer	55.9	68 (35.3)	600	ONSs plus EPA (3.2 g/d)	Standard formula	1	I (4.4), II (25), III (45.6), IV (7.4)
Meng 2021 [10]	China	RCT	Gastric Cancer	59.91	337 (32.3)	100	ONSs	Dietary advice alone	3	I (25.8), II (28.8), III (38.6), IV (6.8)
Miyazaki 2021 [11]	Japan	RCT	Gastric Cancer	66.4	880 (35.6)	400	ONSs	Regular diet	3, 6, 12	I (61.4), II (22.9), III (15.4), IV (0.4)

ONSs, oral nutritional supplements. EPA, eicosapentaenoic acid.

**Table 2:** Effects of moderators of oral nutritional supplements.

Variables	*k*	*β*	MD	95% CIL	95% CIH	*p*
No. of total patients	7	0.000		−0.001	0.002	0.620
Age	7	0.003		−0.098	0.104	0.956
Female rate	7	−2.505		−14.767	9.757	0.689
Duration (month)	7	0.232		−0.150	0.614	0.233
TNM stage						0.019
≥3	2		0.500	0.388	0.611	
<3	5		1.046	0.603	1.488	
Kcal consumption						0.018
Over 400 Kcal	3		0.500	0.389	0.611	
Under 400 Kcal	4		1.081	0.612	1.550	
Country						<0.001
Brazil	1		0.500	0.388	0.612	
Japan	6		0.842	0.758	0.926	
China	1		1.510	1.168	1.852	

*k*, number of effect sizes; β, regression coefficient; MD, mean difference; *p*-value from the meta-regression analysis using the restricted maximum likelihood. CIL and CIH, confidence interval low and high, respectively.

## Data Availability

This study used publicly available data, which are available through the National Health Insurance Service and Health Insurance Review & Assessment Service.

## Supplementary Materials

The following supporting information can be downloaded at: https://www.mdpi.com/article/10.3390/nu15183924/s1, Figure S1. Funnel plots for publication bias. Table S1. Search queries. Table S2. RPISMA checklist.
